# Allergic Inflammation Triggers the Unfolded Protein Response and *Ormdl3* Expression in Rat Adipocytes, While miR-665 Inhibition Selectively Modulates the IRE1/XBP1 Pathway and IL-6 Secretion

**DOI:** 10.3390/ijms27062608

**Published:** 2026-03-12

**Authors:** Joanna Nowakowska-Lewicka, Wojciech Langwiński, Tatiana Wojciechowicz, Marek Skrzypski, Beata Narożna, Maria Kachel, Kosma Sakrajda, Zuzanna Stachowiak, Aleksandra Szczepankiewicz

**Affiliations:** 1 Poznan University of Medical Sciences, Molecular and Cell Biology Unit, Department of Pediatric Pulmonology, Allergy and Clinical Immunology, 60-572 Poznan, Polandksakrajda@ump.edu.pl (K.S.);; 2 Poznan University of Medical Sciences, Doctoral School, 60-812 Poznan, Poland; 3 Poznan University of Life Sciences, Department of Animal Physiology and Biochemistry, Wołyńska 35, 60-637 Poznan, Poland; tatiana.wojciechowicz@up.poznan.pl (T.W.); marek.skrzypski@up.poznan.pl (M.S.); 4 Poznan University of Medical Sciences, Centre of Experimental Medicine, 60-806 Poznan, Poland

**Keywords:** UPR, ER stress, adipocytes, allergic inflammation, miRNA, miR-665

## Abstract

Endoplasmatic reticulum (ER) stress is an imbalance between the load of unfolded proteins and the ability of cellular mechanisms to handle it. Under the influence of this stress, cells activate the unfolded protein response (UPR). The molecular mechanisms of ER stress have been repeatedly linked to metabolic and inflammatory diseases, such as obesity and allergic inflammation. The aim of our study was to investigate if the allergic inflammation in adipocytes affects the expression of UPR pathway genes and Ormdl3 and whether miRNA-665 can modify inflammatory response in adipocytes. We isolated rat preadipocytes and treated them with IL-13 to induce allergic inflammation. Later, we transfected them with miRNA-665 inhibitor. RNA was isolated from adipocytes and analyzed by qPCR. From cell culture medium, we performed an LDH assay and ELISA for secreted IL-6 and TNFα proteins. A comparison between control cells and IL-13-treated cells showed significant differences in the expression of most of the studied UPR pathway genes, Ormdl3 and Bax. Comparing the IL-13-treated cells after miR-665 transfection with non-transfected ones, we observe significant differences only in Ire1α gene. Our research suggests that allergic inflammation induces an adaptive UPR in adipocytes and miR-665 may selectively modify this response, triggering the IRE1/XBP1 axis.

## 1. Introduction

Obesity is a disease of civilization; it is extremely common nowadays and developing at an alarming rate. Adipose tissue, observed in excess in obesity, consists mainly of adipocytes, as well as immune cells, and acts as an active endocrine organ that regulates the metabolism and immune processes of the body by secreting adipokines and inflammatory cytokines (such as TNFα and IL-6) [1]. In healthy conditions, adipose tissue exerts an anti-inflammatory effect, for example through the secretion of adiponectin. However, obesity has the opposite effect—adipose tissue becomes a source of chronic low-grade inflammation, known as metaflammation, caused by the disrupted secretion of over 50 hormones and cytokines [2,3]. An excessive amount of adipose tissue and adipocyte hypertrophy also coexist with chronic inflammatory conditions such as allergic inflammation in asthma. In adipose tissue, activation of the NLRP3 inflammasome leads to caspase-1-dependent maturation of IL-1β and IL-18, which may subsequently contribute to a broader pro-inflammatory cytokine milieu. This inflammatory environment can indirectly promote Th2- and Th17-associated responses, including increased levels of IL-5, IL-13, and IL-17. Metaflammation exacerbates the existing Th2-dependent immune response, leading to a poorer response to steroids and a more severe course of lung disease. Numerous cellular stress pathways contribute to the development of metaflammation [4,5,6]. Adipocyte hypertrophy causes endoplasmic reticulum (ER) stress, which is defined as an imbalance between the load of unfolded proteins and the ability of cellular mechanisms to handle this load [7]. Under the influence of this stress, cells activate a series of adaptive mechanisms known as the unfolded protein response (UPR).

The UPR system can activate adaptive mechanisms and restore homeostasis in the cell or trigger apoptosis, eliminating damaged cells. Apart from restoring homeostasis, previous studies showed that UPR also has many other roles: it regulates metabolism (including lipids and cholesterol) and cell differentiation, and is also significantly involved in inflammatory processes [8]. The UPR pathway includes three main sensors: PERK, ATF6, and IRE1. Each of them activates separate transcriptional and translational factors that partially overlap. IRE1 excises an intron from the mRNA of the transcription factor XBP1, converting it into an active spliced form (XBP1s), to produce chaperones. In addition to its leading role in regulating the adaptive UPR, XBP1 also has important metabolic significance—it facilitates lipid biosynthesis and is essential for the differentiation of B lymphocytes into plasma cells [9]. PERK phosphorylates eIF2α to decrease global protein synthesis, whereas ATF6 undergoes cleavage by proteases in ER, and its fragment enters the nucleus to activate chaperone-encoding genes [10,11]. The molecular mechanisms of ER stress have been repeatedly linked to metabolic and inflammatory diseases [12,13,14], confirming that UPR dysregulation may be involved in many pathologies and may have consequences far beyond the local effects on individual tissues [15].

One of the genes, which is actively involved in the ER stress mechanism, is the ORMDL3 gene, that encodes a transmembrane protein located in the ER. Also, genome-wide association studies (GWASs) showed a strong association between loci on chromosome 17q21 and asthma (including ORMDL3 locus) [16,17,18]. Thus, the ORMDL3 gene may be a bridge linking the gap between genetic susceptibility to asthma and UPR activation in response to inflammatory signals [19,20].

ER stress and the UPR play a key role in the pathogenesis of asthma, as one of the chronic inflammatory diseases [21,22]. One of the key Th2 cytokines in atopic asthma is IL-13, which acts on airway epithelial cells, causing mucus overproduction, remodeling, and bronchial hyperresponsiveness. Its administration into the airways is sufficient to induce airway hyperresponsiveness (AHR), eosinophilia, and increased mucus production, while the IL-13 blockade significantly inhibited all these effects in animal models of allergic asthma [23,24]. ER stress is an integral mechanism that controls inflammation, cell apoptosis, and structural changes in the airways, acting through all three main branches of the UPR pathway. This cytokine contributes to the activation of ER stress and UPR-activated inflammatory pathways [25], thus IL-13 may represent a critical link between allergic inflammation and ER stress-related pathways, not only in the airways but also in metabolically active cells such as adipocytes [26].

Recent evidence suggests that miRNAs also play a key role in inflammatory processes and the cellular ER stress pathway in adipose tissue [27]. One of them, miR-665, has been shown to inhibit the expression of two key components of the UPR signaling pathway—XBP1 and ORMDL3 [28]. These studies suggest a possible role for miR-665 in regulating inflammatory and ER stress response; however, its influence on the adipose tissue upon allergic inflammation remains unexplored. Our goal was to investigate whether the allergic inflammation in adipocytes affects the expression of UPR pathway genes and *Ormdl3*, and whether potential regulators of its activity, such as miRNA-665, can modify inflammatory response in adipocytes.

## 2. Results

### 2.1. Confirmation of the Differentiation of Preadipocytes into Adipocytes

**Figure 1:**
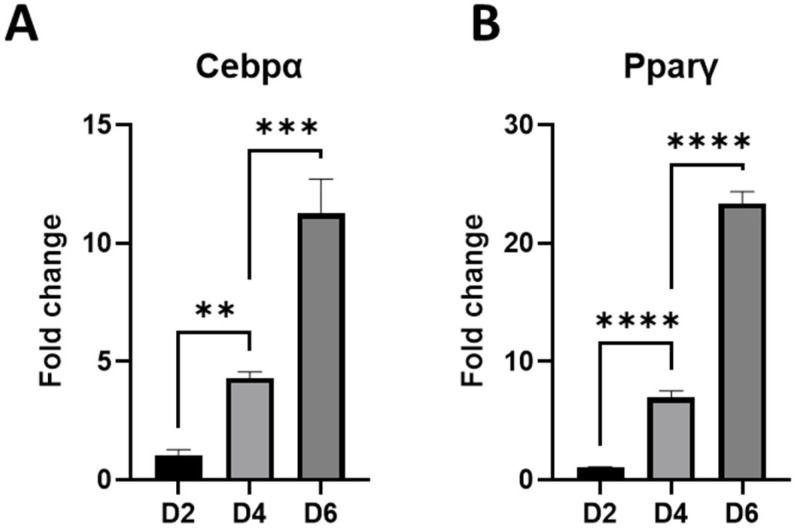
The expression of Cebpα and Pparγ genes increases in maturing adipocytes. All analyzed time points differed from each other (one-way ANOVA, Tukey’s post hoc test, n = 3 samples per group). The largest difference was observed between D2 and D6 (*p* < 0.0001 for both genes), while for clarity, the graph shows comparisons between successive measurement points. (**A**)—for the Cebpα gene: D2 vs. D4 (*p* = 0.0082), D4 vs. D6 (*p* = 0.0001). (**B**)—for the Pparγ gene: D2 vs. D4 (*p* < 0.0001), D4 vs. D6 (*p* < 0.0001). Fold change is presented in relation to Gapdh; (** for *p* < 0.005), *** for *p* < 0.0005, **** for *p* < 0.00005).

To confirm that the rat preadipocytes differentiated into mature adipocytes in vitro after adding hormones, we analyzed the expression of Cebpα and Pparγ genes in control cells collected on days 2, 4, and 6 of culture (D2, D4, D6). We observed significantly increased expression of these genes over time, confirming the cell differentiation (Figure 1).

### 2.2. LDH Release Analysis Confirms Lack of Cytotoxic Effects of Il-13 and miR-665 Inhibition in Adipocytes

**Figure 2:**
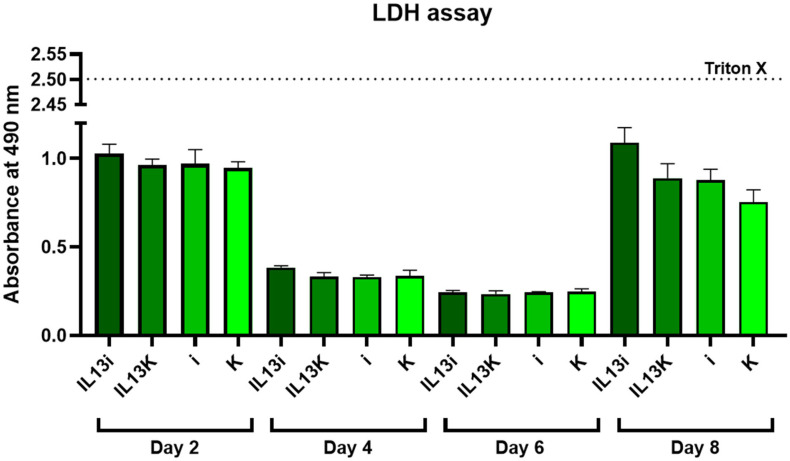
LDH assay confirms lack of cytotoxicity of IL-13 and miR-665 in adipocytes. Representative LDH release in the culture medium on days 2, 4, 6 and 8. Each bar represents mean value ± SD, one-way ANOVA test, n = 4 samples per group.

We also confirmed that neither Il-13 nor miR-665 inhibitor causes cytotoxic effects in adipocytes by comparing the culture medium material from all experimental groups (IL-13i, IL-13C, i and C) throughout the course of the experiment (D2, D4, D6, and D8) (Figure 2).

### 2.3. IL-13 Induces Activation of Three Major UPR Pathways in Mature Adipocytes

**Figure 3:**
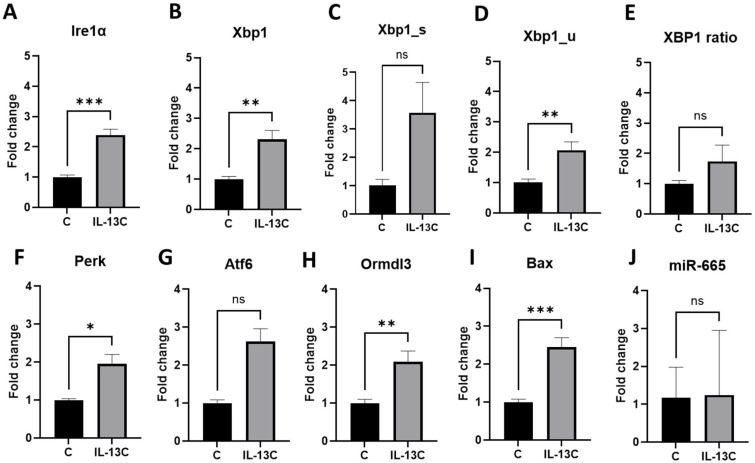
Increased expression of UPR pathway genes, *Ormdl3*, *Bax* and miR-665 after stimulation with IL-13. Comparative expression analysis between cells treated with IL-13 (IL-13C) and non-treated control cells (K) for the genes from the IRE1α signaling pathway: (**A**): Ern1 (Ire1α) (*p* = 0.0003, unpaired *t*-test), (**B**): Xbp1 (*p* = 0.0019, unpaired *t*-test), (**C**): Xbp1_s—spliced (*p* = ns, Mann–Whitney test), (**D**): Xbp1_u—unspliced (*p* = 0.0038, unpaired *t*-test), (**E**): Xbp1 ratio (*p* = ns, unpaired *t*-test), for the Eif2ak3 (Perk) gene: (**F**) (*p* = 0.0204, Welch’s test) for the Atf6 gene: (**G**) (*p* = ns, Mann–Whitney test), for the Ormdl3 gene: (**H**) (*p* = 0.0034, unpaired *t*-test), for the Bax gene: (**I**) (*p* = 0.0006, unpaired *t*-test), for miR-665: (**J**) (*p* = ns, Mann–Whitney test). n = 3 samples per each group. Fold change is presented in relation to Gapdh (**A**–**I**) or miR-26a (**J**). (ns = non-significant, * for *p* < 0.05, ** for *p* < 0.005, *** for *p* < 0.0005).

We analyzed the expression of genes of three main UPR pathways (Ern1, Eif2Ak3, and Atf6) upon the allergic inflammation stimulated by IL-13. We also analyzed the expression of XBP1 (total, spliced, and unspliced forms), a downstream target of IRE1, due to its association with miR-665. In qPCR, we observed the significantly higher expression of most of these genes in the preadipocytes that received IL-13 on D2 and D4 (IL-13C) compared to the untreated cells from the control group (C) (Figure 3A–G).

To better evaluate the activation of the IRE1 pathway, the ratio of spliced to unspliced XBP1 (XBP1s/XBP1u) was calculated for each sample. IL-13 stimulation increased the XBP1s/XBP1u ratio compared to the control, indicating activation of the adaptive UPR branch (Figure 3E).

We were also interested in how the expression of the Ormdl3 gene, linking asthma, UPR, and miR-665, would develop. We observed increased Ormdl3 expression in cells treated with IL-13 (IL-13C) in comparison to control cells (C) (Figure 3H).

Increased UPR genes and Ormdl3 expression confirmed that after adipocytes stimulated with IL-13 undergo allergic inflammation, they induced ER stress. To determine the effect of this stress, we analyzed the expression of the Bax gene, which initiates the proapoptotic signal in the cell. We observed its significantly increased expression in IL-13-treated cells (IL-13C) compared to control cells (C) (Figure 3I).

When analyzing miR-665 expression between these groups, we observe a continuation of the trend of higher expression in the IL-13C group compared to the C group (Figure 3J).

### 2.4. Confirmation of Transfection with miR-665 Inhibitor

**Figure 4:**
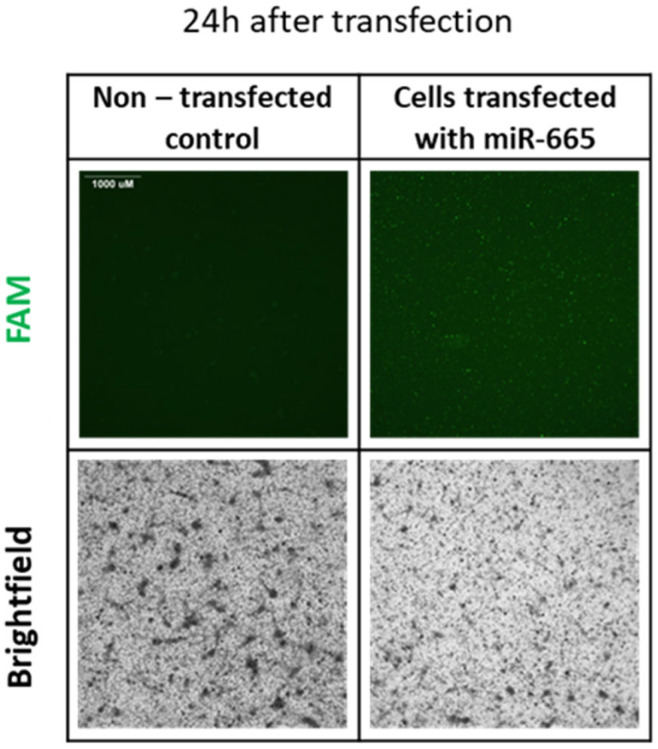
The microscopic photographs showing the transfection of mature adipocytes with miR-665 inhibitor. Representative bright- and widefield fluorescent imaging 24 h after transfection, 4× objective, FAM dye (laser: 15, exposure time: 150 ms). Scale bar: 1000 μm.

Transfection of mature adipocytes with miR-665 inhibitor labeled with FAM at a concentration of 50 nM was performed on the sixth day of the experiment (D6) using an INTERFERin. We confirmed the transfection after 24 h, comparing the transfected cells with the non-transfected group (Figure 4). Due to the fact that FAM is a dye susceptible to photobleaching, the effect of transfection was more visible 24 h after transfection, and after 48 h the visibility of the dye significantly weakened.

### 2.5. Selective Modulation of the IRE1/XBP1 Axis by miR-665

**Figure 5:**
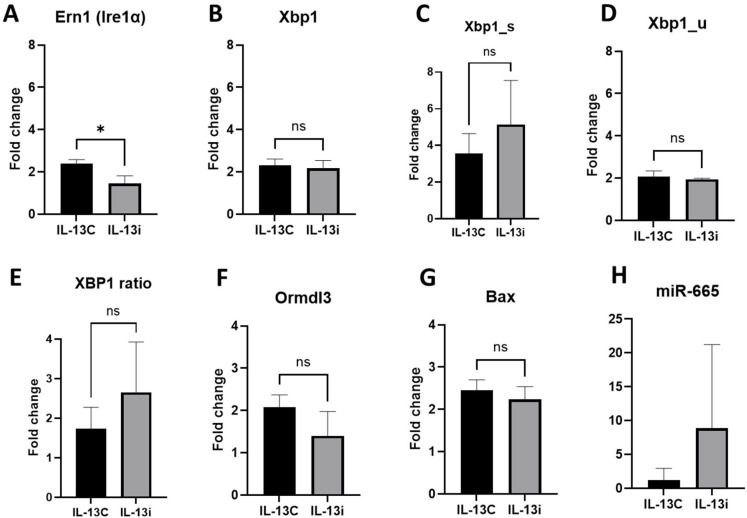
Decreased expression of most of the IRE1α signaling pathway genes, *Ormdl3*, *Bax* and *miR-665,* after stimulation with IL-13 and miR-665 inhibitor transfection. Comparative expression analysis between cells treated with IL-13 (IL-13C) and cells treated with IL-13 and then transfected with miR-665 (IL-13i) for the genes from the IRE1α signaling pathway: (**A**): Ern1 (Ire1α) (*p* = 0.0165, unpaired *t*-test), (**B**): Xbp1 (*p* = ns, unpaired *t*-test), (**C**): Xbp1_s—spliced (*p* = ns, Mann–Whitney test), (**D**): Xbp1_u—unspliced (*p* = ns, unpaired *t*-test), (**E**): Xbp1 ratio (*p* = ns, Mann–Whitney test), for the Ormdl3 gene: (**F**) (*p* = ns, unpaired *t*-test), for the Bax gene: (**G**) (*p* = ns, unpaired *t*-test), for miR-665: (**H**) (*p* = ns, Mann–Whitney test). n = 3 samples per each group. Fold change is presented in relation to Gapdh (**A**–**G**) or miR-26a (**H**). (ns = non-significant, * for *p* < 0.05).

To examine how miR-665 affects adipocytes after induction of allergic inflammation, we compared cells treated with IL-13 on day 2 and day 4 of the experiment (IL-13C) with cells also treated with IL-13 on day 2 and day 4 of the experiment that were transfected with miR-665 on day 6 of the experiment (IL-13i). We observed significant changes only in the IRE1/XBP1 pathway—cells transfected with the miR-665 inhibitor showed significantly lower Ern1 (Ire1) gene expression than non-transfected cells; however, the effect on the expression of *Xbp1* was not significant (Figure 5A–D). Inhibition under inflammatory conditions increased the XBP1s/XBP1u ratio compared to IL-13 alone, suggesting differential regulation of IRE1α signaling at the transcriptional and post-transcriptional levels (Figure 5E). Considering both Ormdl3 and Bax genes, we also did not observe any significant expression changes between those groups (Figure 5F,G). We also examined miR-665 expression, which did not significantly differ between groups. However, inhibition of IL-13-induced signaling resulted in a marked increase in miR-665 levels compared to IL-13 treatment alone (Figure 5H).

### 2.6. Differential Regulation of IL-6 and TNFα Secretion in Adipocytes Under Allergic Inflammation Conditions

**Figure 6:**
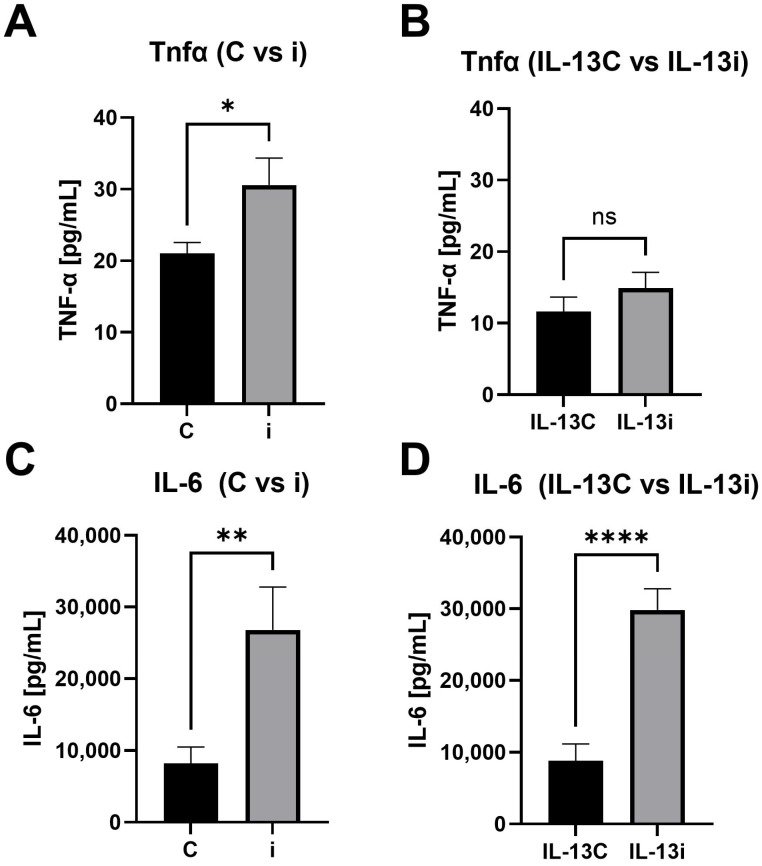
Increased concentrations of TNFα and IL-6 on day 8 of the experiment upon miR-665 inhibitor transfection. Comparative analysis of TNFα secretion between control cells (C) and miR-665 transfected cells (i) on day 8 of the experiment: (**A**) (*p* = 0.0286, Mann–Whitney test) and between IL-13-treated cells with (IL-13i) and without miR-665 inhibition (IL-13C): (**B**) (*p* = ns, Welch’s test). Comparative analysis of IL-6 secretion between control cells (C) and miR-665 transfected cells (i) on day 8 of the experiment: (**C**) (*p* = 0.0048, Welch’s test) and between IL-13-treated cells with (IL-13i) and without miR-665 inhibition (IL-13C): (**D**) (*p* < 0.0001, Welch’s test). n = 4 samples per each group. (ns = non-significant, * for *p* < 0.05, ** for *p* < 0.005, **** for *p* < 0.00005)

To evaluate changes in inflammatory cytokine secretion, we measured the concentrations of TNFα and IL-6 in culture supernatants at day 8 of the experiment (48 h post-transfection). Comparisons were performed between control (C) and miR-665 transfected cells (i), as well as between IL-13-treated cells with (IL-13i) or without miR-665 inhibition (IL-13C). We showed that the transfection with miR-665 inhibitor increased the levels of both cytokines compared to the non-transfected groups (Figure 6A–D).

## 3. Discussion

In our work, we focused on the effect of Th2-type allergic inflammation induced by IL-13 on preadipocytes differentiated into adipocytes, as they are highly active participants in immunometabolic networks, controlling not only energy homeostasis but also specific immune responses [29]. The present study focused primarily on the adaptive IRE1/XBP1 branch of the UPR. CHOP, predominantly associated with sustained PERK–eIF2α–ATF4 activation and pro-apoptotic ER stress, was not included, as the experimental model was designed to assess early adaptive responses rather than terminal ER stress-induced apoptosis. Our results showed that allergic inflammation triggered by IL-13 activates two main pathways of the UPR system in adipocytes and this is the first study that demonstrated the influence of allergic inflammation on UPR in adipocytes. Previous studies showed that IL-13 has been previously linked to UPR in the bronchial epithelium, where IL-13 stimulated the activation of STAT6 and subsequently ORMDL3 expression and enhanced ER stress in these cells. The activation of UPR pathways was also observed in patients with severe eosinophilic asthma, suggesting that ER stress and UPR may be driven by allergic inflammation in the airways [30,31]. In this study, we observed that this link is also observed in adipocytes where allergic inflammation, induced by IL-13, drives cellular ER stress and activates UPR. Our results suggest that Th2 cytokines increase ER stress via increased expression of UPR genes, as well as *Ormdl3*, a ER stress-related gene. Another interesting observation was the significantly increased expression of the proapoptotic *Bax* during allergic inflammation, suggesting that UPR activation induced proapoptotic signals in adipocytes.

Our previous results showed significantly increased expression of miR-665 in adipose tissue in the rat model of asthma induced by house dust mite exposure (FC = 12,5; *p* = 0.013). Therefore, taking into account that this miRNA was shown to target the *Xbp1* and *Ormdl3* genes, we aimed to analyze if miR-665 inhibition may influence UPR pathway response during allergic inflammation. We demonstrated that miR-665 inhibition selectively influences the expression of the Ire1α gene from the UPR system, but not other analyzed genes in rat adipocytes during allergic inflammation. Thus, our data indicate that in the presence of allergic inflammation, the miR-665 inhibitor may have a more selective effect, mainly through the Ire1/Xbp1 UPR axis. However, since only one gene of the UPR pathway showed enhanced expression in the presence of miR-665 inhibitor and allergic inflammation, without activation of the other genes or the proapoptotic *Bax*, we can suggest that it may exert a protective effect against ER stress and the subsequent activation of the UPR pathway and initiation of apoptosis in adipocytes under allergic inflammatory conditions.

An interesting observation is the increase in IL-6 secretion from adipocytes during and after miR-665 inhibitor transfection either in the absence or presence of allergic inflammation. This observation suggests that miR-665 inhibition did not prevent inflammatory cytokine secretion from adipocytes independent of stimulation with IL-13. Previous studies showed that the role of IL-6 secreted by adipocytes does not trigger the classical inflammatory cascade, but rather leads to post-transcriptional regulation and affects the metabolism of other cells. Interestingly, the inflammatory response in adipocytes also depends on the source of IL-6 [32,33].

TNFα secretion also increased after miR-665 inhibition, but interestingly only in the absence of allergic conditions triggered by IL-13. This may suggest that IL-13 significantly affects the profile of cytokines secreted by adipocytes, which may mask standard pro-inflammatory responses—as in this case—masking TNFα secretion. Furthermore, the main sources of TNFα in adipose tissue are macrophages, which emphasizes that the adipocytes can modulate what is secreted; however, we would need immune cells in the model to observe the whole picture [34].

We are aware of the several limitations of our study. First of all, we worked on an in vitro model, so we could not take into account the influence of immune cells on adipocytes, which would be possible in an in vivo model. While an in vitro approach minimizes systemic confounding factors, in vivo studies will be necessary to validate the physiological relevance of these findings in complex metabolic–inflammatory settings. We also do not know the exact mechanism of action of miR-665; we lack confirmation regarding whether it targets one of the genes in the signaling pathway or acts indirectly by influencing other genes regulating ER stress and unfolded protein response.

Although our findings provide initial evidence linking IL-13-associated inflammatory signaling with adaptive ER stress responses in adipocytes, the precise molecular mechanisms underlying this interaction require further investigation. Future studies should address whether this crosstalk operates through direct transcriptional regulation, miRNA-mediated modulation, or broader metabolic–inflammatory feedback loops.

## 4. Materials and Methods

### 4.1. Animals, Preadipocyte Isolation and Culture: Transfection

#### 4.1.1. Rats

Preadipocytes were obtained from eight Wistar male rats, weighing 100–200 g, at the age of 10–14 weeks. Animals were fed ad libitum with standard chow and maintained in a 12:12 light–dark cycle. Rats were sacrificed by decapitation.

Adipose tissue samples were obtained from animals sacrificed for purposes unrelated to the present study. No experimental procedures were performed on live animals within the scope of this work. According to Polish regulations and the guidelines of the Local Ethical Committee, ethical approval was not required.

#### 4.1.2. Preadipocyte Isolation

Rat white fat precursor cells were isolated from epididymal adipose fat pads, as described in our previous study [35].

#### 4.1.3. Preadipocyte Culture and Differentiation

Preadipocytes were seeded in 12-well plates and cultured under the standard conditions (37C, 5%CO_2_) in DMEM/F12 medium, supplemented with 10% FBS and antibiotics. After 24 h, the medium was changed to the differentiation one (DMEM/F12 with 0.1% BSA without fatty acids, 167 nM insulin and 2 nM triiodothyronine). The composition of the medium remained unchanged until the end of the experiment; we replaced it every 2 days. On days 2 and 4 of the experiment, Il-13 was added to the culture medium at a concentration of 80 ng/mL.

The cells were divided into 4 groups:

- IL-13i—cells treated with IL-13 on day 2 and day 4 and then transfected with miR-665 inhibitor on day 6.
- IL-13C—cells treated with IL-13 on day 2 and day 4.
- i—cells transfected with miR-665 inhibitor on day 6.
- C—control cells not transfected and not treated with IL-13.

Sometimes in the text, days are presented in abbreviated form: D2, D4, D6 and D8.

#### 4.1.4. miRNA Transfection

Transfection was performed with INTERFERin transfection reagent (Polyplus transfection, Illkirch, France) according to the manufacturer’s recommendation. The transfection mixtures were prepared in OptiMem (Thermo Fisher Scientific, Waltham, MA, USA). A complete culture medium was added to each well to obtain a total volume of 1 mL/well. The molecule used for the transfection was rno-miR-665 (5′ACCAGGAGGCUGAGGUCCCUUA3′) (Thermo Fisher Scientific, Waltham, MA, USA), labeled with FAM on 5′ end, at a concentration of 50 nM. Transfection was performed on day 6 of the experiment.

### 4.2. Lactate Dehydrogenase Assay (LDH)

Lactate dehydrogenase activity (Cytotoxicity detection kit LDH, Sigma-Aldrich, St. Louis, MO, USA) was assessed every 2 days in secreted supernatants, according to the manufacturer’s protocol. Prior to use, the collected supernatants were diluted in DMEM-F12 medium (50:50 *v*/*v*). Absorbance at 490 nm was measured using a microplate reader (ASYS UVM 340 (Biocompare, South San Francisco, CA, USA)), with 620 nm as a reference, against a background control (i.e., DMEM-F12 medium). Separate control cells were treated with a 2% Triton X-100 solution for 30 min to induce total cell lysis and maximal LDH release.

### 4.3. Fluorescent Microscope Imaging

Rat adipocytes were imaged in real time 24 h and 48 h after transfection using a 4× objective. A conventional inverted fluorescence microscope (Nikon Ts2R (Nikon Instruments, New York, NY, USA)) was used, employing the brightfield and green fluorescence (GFP) channels. The specific microscope settings are detailed in the corresponding figure legend.

### 4.4. RNA Analysis

Forty-eight hours after transfection, on day 8 of the experiment, all cells were harvested and placed in 400 μL of MiRLys buffer supplemented with β-mercaptoethanol and stored at −80 °C prior to isolation. The RNA and miRNA were isolated using ExtractMe Kit (Blirt, Gdańsk, Polska). For reverse transcription, 300 ng of RNA was used along with the GoScript Reverse Transcription Kit (Promega, Madison, WI, USA) and 0.5 ng of miRNA with the TaqMan Advanced miRNA cDNA synthesis kit (Thermo Fisher Scientific, Waltham, MA, USA). The relative expression analysis was conducted using quantitative reverse transcription PCR (qRT-PCR) and GoTaq qPCR Master Mix (Promega), employing specific primers for the following genes: Cebpα, Pparγ, Ern1, Xbp1, Xbp1_s, Xbp1_u, Eif2ak3, Atf6, Ormdl3, Bax, Gapdh (Table A1, Appendix A). All calculations were performed using the delta–delta Ct method, with Gapdh serving as the reference gene. The qPCR reaction for miRNA was conducted with TaqMan Fast Advanced Master Mix and TaqMan Advanced miRNA Assays for miR-665 and miR-26a-5p.

### 4.5. Measurement of Proteins Secreted to the Culture Medium—ELISA

The culture medium from above the cells was collected every 2 days (each time the medium was changed) and stored at −80 °C prior to further analysis. The levels of IL-6 and TNFα were measured using Rat DuoSet ELISA kits (Bio-Techne, Minneapolis, MN, USA), according to manufacturer’s protocols. For TNFα, the medium was undiluted; for IL-6, the medium was diluted 1:10. The absorbance was read on a plate reader at a wavelength of 450 nm (Asys UVM 340). Protein concentration was quantified against a standard curve calibrated with known amounts of protein.

### 4.6. Statistical Analysis

Statistical analyses were performed using GraphPad Prism 10 software. The normality of the distribution was tested using the Shapiro–Wilk test. All study variables were independent; as such, for comparisons between two groups, Student’s *t*-tests for independent groups were performed. For comparisons between more than two groups, one-way analysis of variance (ANOVA) or two-way ANOVA for independent groups was performed. The homogeneity of variance was checked using the Fisher–Snedecor test. For groups that did not show equal variances, Student’s t-test was performed with Welch’s correction or ANOVA, together with the Brown–Forsythe test and Tukey’s post hoc test. A value of *p* < 0.05 was considered statistically significant. According to the order of magnitude for *p*-values, analyses were marked with asterisks (* for *p* < 0.05, ** for *p* < 0.005, *** for *p* < 0.0005, **** for *p* < 0.00005).

## 5. Conclusions

Our research suggests that IL-13 induces an adaptive UPR, confirming that during allergic inflammation, ER stress response is significantly affected in adipocytes. We also showed that miR-665 modifies this response; in the presence of its inhibitor and allergic inflammation, we observed a response only in the Ire1α gene, suggesting that it may selectively influence the modulation of this sensor of the UPR pathway. Further research is necessary to better understand this possible link between allergic inflammation, UPR and adipocytes.

## Data Availability

All raw and processed data will be made available upon reasonable request to the corresponding author.

## Appendix A

**Table A1:** qPCR rat primer sequences.

Rat Gene Name	Primer F (5′-3′)	Primer R (5′-3′)
*Cebpα*	ATAAAGCCAAACAGCGCAAC	CGGTCATTGTCACTGGTCAA
*Pparγ*	CAGGAAAGACAACAGACAAATCA	GGGGGTGATATGTTTGAACTTG
*Ern1* (*Ire1α*)	GTTGCTGTGGAGACCCTACG	CACATACAGAGTGGGCGTCA
*Xbp1*	CACAGACTGCGCGAGATAGA	AGCTGGAGTTTCTGGTTCTCT
*Xbp1_s*	TGCTGAGTCCGCAGCAGG	ACAGGGTCCAACTTGTCCAG
*Xbp1_u*	GTCCGCAGCACTCAGACTAC	TGCCCAAAAGGATATCAGACTCA
*Eif2ak3* (*Perk*)	GCTACACACACGGGACAAGT	GGAGTAGTTGTTTCCATGAATCTGTT
*Atf6*	CACGACAGAGTCTCTCAGGTTG	TCAGCTGAGAATTCGAGCCC
*Ormdl3*	ACCTTATCCATAACCTGGGCA	TACAGCACAATGGGCGTGAT
*Bax*	CATCCACCAAGAAGCTGAG	CAGCAATCATCCTCTGCAG
*Gapdh*	AACTCCCTCAAGATTGTCAGCAA	GGCATGGACTGTGGTCATGA
